# AGING AND MITOCHONDRIAL DISEASE: SHARED MECHANISMS AND THERAPIES?

**DOI:** 10.1093/geroni/igz038.1459

**Published:** 2019-11-08

**Authors:** Alessandro Bitto, Herman Tung, Kejun Ying, Daniel L Smith, Ernst-Bernhard Kayser, Philip G Morgan, Margaret M Sedensky, Matt Kaeberlein

**Affiliations:** 1 University of Washington, Seattle, Washington, United States; 2 Allen Institute for brain science, Seattle, Washington, United States; 3 School of Life Sciences, Sun Yat-sen University, Guangzhou Shi, Guangdong, China (People’s Republic); 4 Department of Nutrition Science, University of Alabama, Birmingham, Alabama, United States; 5 Seattle Children’s Research Institute, Seattle, Washington, United States; 6 Department of Pathology, University of Washington, Seattle, Washington, United States

## Abstract

Mitochondrial disease describes multiple pathologies characterized by a wide array of disease symptoms and severity, caused by mitochondrial dysfunction in one or multiple organs. Aging organisms display a similar variety of disease phenotypes, which are often characterized by mitochondrial impairment. Despite the heterogeneity of aging phenotypes, several interventions have been identified which can increase lifespan and delay the onset of age-related diseases in multiple organisms. Two age-delaying interventions, rapamycin and acarbose, dramatically suppress pathology in a mouse model of mitochondrial disease caused by depletion of the NADH-Ubiquinone Oxidoreductase Complex (Ndufs4-/-). This model recapitulates human Leigh syndrome, a childhood mitochondrial disease. Upon treatment with either drug, disease suppression is accompanied by a remodeling of nutrient metabolism and restoration of the NAD+/NADH ratio in the brain without affecting the electron transport chain. Thus, we propose that metabolic derangements induced by mitochondrial dysfunction may be a shared mechanism of aging and mitochondrial disease.

